# From fork to fatherhood: Unveiling the link between dietary pattern indices and biochemical parameters of semen

**DOI:** 10.1371/journal.pone.0338878

**Published:** 2026-05-06

**Authors:** Ali Taheri Madah, Nargesbano Jahangiri, Mahtab Dabagh, Sahar Rostami, Fardin Amidi, Mahshad Khodarahmian, Masoome Jabarpour, Ali Tavoosian, Asma Kheirollahi, Maryam Shabani Nashtaei, Akram Vatannejad

**Affiliations:** 1 Department of Anatomy, School of Medicine, Tehran University of Medical Sciences, Tehran, Iran; 2 Department of Infertility, Yas Hospital Complex, Tehran University of Medical Sciences, Tehran, Iran; 3 Department of Gynecology and Obstetrics, Arash Women’s Hospital, Tehran University of Medical Sciences, Tehran, Iran; 4 Department of Infertility, Shariati Hospital, Tehran University of Medical Sciences, Tehran, Iran; 5 Department of Urology, School of Medicine, Tehran University of Medical Sciences, Tehran, Iran; 6 Department of Comparative Biosciences, Faculty of Veterinary Medicine, University of Tehran, Tehran, Iran; King Saud University / Zagazig University, EGYPT

## Abstract

A cross-sectional design was employed to elucidate the interplay of dietary pattern index scores and seminal and serum biochemical parameters in men presenting to our infertility clinics. Ninety men with idiopathic infertility were enrolled. Semen was collected and evaluated following the World Health Organization (WHO) 2010 guideline for semen analysis (SA). Participants’ dietary habits were evaluated using a 168-item semi-quantitative food frequency questionnaire (FFQ). Independent samples t-tests were performed to compare groups with normal and abnormal semen quality parameters in terms of demographic characteristics, energy intake, and initial semen analysis results. The association between dietary scores—the Alternative Healthy Eating Index (AHEI), the dietary total antioxidant capacity (dTAC), and the dietary inflammatory index (DII)—and semen parameters, specifically total antioxidant capacity (TAC) and tumor necrosis factor-alpha (TNF-α) levels, was investigated using one-way analysis of variance (ANOVA) followed by Tukey’s post hoc tests for multiple comparisons. Higher intake of advanced glycation end products (AGE) was associated with abnormal semen parameters (p = 0.04). No other significant differences in serum/semen biochemistry were detected (all p-values > 0.05). There were no associations between dTAC, AHEI, and DII scores, and TAC or TNF-α (all p-values > 0.05). However, AHEI quartiles showed significant differences in seminal AGE levels (p = 0.002). Semen malondialdehyde (MDA) levels were higher in the abnormal semen group (1.03 ± 0.28 vs. 0.95 ± 0.22; p = 0.09). No associations were observed between dTAC or DII scores and oxidative stress markers (all p-values > 0.05). AHEI quartiles showed a similar pattern in serum MDA and AGE levels. This study adds to the evidence regarding the dietary impact on male fertility.

## Introduction

With a male factor being the single, definitive cause in 20−30% of cases and a contributing cause in a further 20%, infertility continues to pose a major global issue affecting approximately 8−12% of couples [1]. Male infertility is a multifactorial condition arising from a complex interplay of genetic, lifestyle, environmental, and health-related factors that can impair sperm production, maturation, and accessory gland function, ultimately leading to abnormal semen parameters [2]. The prevalence of inflammatory infertility might be underestimated, as immune cell infiltration is often detected only in testicular biopsies from azoospermic individuals [3]. Inflammation impairs sperm function, steroidogenesis, and spermatogenesis [4]. Inflammatory cytokines and chemokines in the testes can disrupt normal function, leading to impaired sperm production, reduced sperm quality, and increased DNA damage [5–7]. Moreover, the male reproductive system experiences inflammatory damage resulting from prolonged oxidative stress (OS), leading to an excessive generation of reactive oxygen species (ROS) by cytokines [8]. Elevated levels of ROS, frequently detected in the ejaculate of infertile men, can potentially compromise sperm membrane integrity through various mechanisms, including lipid peroxidation, alterations in membrane fluidity, oxidative DNA damage, and the formation of advanced glycation end products (AGE) [9]. The primary byproduct of lipid peroxidation, malondialdehyde (MDA), provides a critical indicator of the degree of OS experienced by cell membranes [10]. MDA has been shown to have mutagenic properties, resulting in detrimental effects on sperm DNA integrity and inducing modifications in sperm cell proteins, ultimately leading to a decline in fertility rates [11,12]. Additionally, 8-hydroxy-2’-deoxyguanosine (8-OHdG), a byproduct arising from the oxidation of guanosine residues within DNA, serves as a highly sensitive marker for evaluating DNA damage attributed to excessive ROS. These consequences ultimately reduce fertility and negatively affect semen parameters [13].

There has been a growing interest in exploring the influence of dietary patterns and nutrient intake on male reproduction [14]. Research has demonstrated that the Western diet negatively affects male fertility [15,16], whereas adherence to a balanced, nutritious diet improves sperm quality and reduces the risk of abnormalities [17]. The dietary total antioxidant capacity (dTAC) serves as a proxy for the diet’s antioxidant potential, closely linked to a healthy dietary pattern [18]. The dTAC reflects the overall antioxidant potential of a diet, which is crucial for counteracting OS linked to sperm dysfunction [19]. Several widely used and validated dietary quality indices, including the Healthy Eating Index (HEI) aligned with national dietary guidelines, the Alternative Healthy Eating Index (AHEI) emphasizing components linked to chronic disease prevention, the alternate Mediterranean Diet score (aMED), and the Dietary Approaches to Stop Hypertension (DASH) score, are employed to assess overall dietary adherence. These indices accurately reflect the degree to which individuals adhere to dietary patterns linked to a range of positive health outcomes [20–23]. A growing body of literature has examined the associations between dietary patterns, as measured by HEI, aMED, AHEI, and DASH, and semen characteristics [24–26]. Among these, AHEI-2010 has shown the most significant positive effect on sperm quality [27]. The Dietary Inflammatory Index (DII), a metric incorporating six inflammatory markers linked to dietary elements such as vitamins, minerals, macronutrients, flavonoids, and specific foods [28–30], has been shown to correlate with various long-term health conditions, including metabolic syndrome, cardiovascular disease (CVD), and cancer [31–33]. Chronic inflammation has been linked to impaired spermatogenesis [4]. DII quantifies the potential inflammatory impact of dietary intake, where higher scores indicate a more pro-inflammatory diet [34]. Evidence suggests that the DII might impact men’s reproductive health, with diets high in inflammatory components potentially increasing the risk of low testosterone levels [35]. However, conflicting findings exist regarding the association between DII and specific indicators of male fertility [36,37]. Our previous study on infertile men with normal and abnormal semen parameters [38] revealed no significant differences in semen parameters across quartiles of dTAC, AHEI, and DII scores. However, a trend was observed where men in the highest DII quartile had a higher likelihood of abnormal semen, whereas dTAC and AHEI showed no significant associations. The relationship between dietary patterns and the biochemical composition of semen remains an area of ongoing investigation.

Despite increasing evidence linking dietary quality indices to semen characteristics, the relationship between these indices and seminal biochemical markers of inflammation and oxidative stress remains poorly understood. Most previous studies have focused solely on semen parameters, neglecting underlying molecular mediators that may bridge diet and sperm function. To address this gap, we conducted a cross-sectional study to investigate the associations of dTAC, AHEI, and DII with TNF-α, AGE, total antioxidant capacity (TAC), and 8-OHdG levels in both serum and seminal plasma of infertile men. This integrative approach provides a more comprehensive understanding of how dietary quality and inflammatory potential relate to oxidative and inflammatory markers involved in male reproductive function.

## Methods

### Patient recruitment and study design

We examined associations between dietary quality scores and serum and seminal biochemical parameters among 90 infertile men. Participants were recruited from the Infertility Clinics of Dr. Shariati and Arash Women’s Hospitals, Tehran, Iran, from November 26, 2020, to October 10, 2022. All patient data were anonymized to ensure confidentiality. Unique codes were assigned to each patient, and no identifying information was accessible during data analysis. The anonymized data were subsequently accessed for research purposes. Participants were infertile men aged 20–40 years, classified according to the WHO (2010) criteria [39], who were unable to conceive, had normal or abnormal semen parameters, showed no reproductive or systemic medical disorders, had normal physical examination results, and did not follow any strict or long-term dietary restrictions. We classified infertility as idiopathic based on a complete workup that identified no specific cause. Male infertility cases with azoospermia and couples experiencing female-associated infertility were not included. All examinations were conducted by an experienced andrologist.

### Demographic and dietary data collection

We collected data on the participants’ demographics, including their body mass index (BMI), age, waist-to-hip ratio (WHR), educational level, smoking status, and supplement intake. The dietary assessment methodology was previously described [38]. In brief, the Tehran Lipid and Glucose Study’s (TLGS) 168-item semi-quantitative food frequency questionnaire (FFQ) [40] was administered to assess participants’ dietary habits over the past year. The questionnaire, validated by Esfahani et al., [41] was administered by trained interviewers and included options for individuals to indicate their usual intake frequency for each food item. If none of the listed frequency options applied, participants reported their portion sizes, which were then standardized. The software Nutritionist IV (First Databank Division, Hearst Corporation, San Bruno, CA, USA) was used to assess food intake, including Iranian foods, and to calculate the usual daily intake (in grams) of each food item.

### Assessment of diet scores (dTAC, DII, and AHEI)

In brief, dietary data collected through the 168-item semi-quantitative FFQ was utilized to compute AHEI, DII, and dTAC, scores for all participants. The DII score, designed to assess the inflammatory potential of an individual’s diet, effectively reflects its influence on various inflammatory biomarkers. This score evaluates the total inflammatory potential of the dietary pattern [42]. The dTAC was calculated by multiplying the daily consumption of each selected food item by its corresponding antioxidant value per serving and then summing these products. Antioxidant contributions from supplements were excluded from the calculation [43]. The Alternative Healthy Eating Index (AHEI) assigns scores to various dietary components based on their potential health advantages, where higher scores reflect a more healthful dietary pattern. The scoring system incorporates components such as fruits, vegetables, nuts, legumes, and whole grains, along with polyunsaturated (PUFAs) and omega-3 fatty acids, while accounting for the intake of sugary drinks, fruit juice, red and processed meats, and sodium [44]. Previous research has established a correlation between diets that receive low scores on the AHEI [21,45,46] and the dTAC [47–49], as well as high scores on the DII [50,51], and the increased risk of numerous long-term health conditions.

### Semen collection, preparation, and analysis

Male patients who had failed to achieve pregnancy after one year of regular, unprotected intercourse were evaluated by an andrologist at our fertility clinics. Before enrollment, patients provided written consent, granting permission to utilize a portion of their semen for research purposes. Fresh semen samples were collected through masturbation into sterile polypropylene containers following 3–5 days of sexual abstinence. Samples were delivered to the laboratory within one hour of collection. Upon receipt, samples were incubated at 37°C for 30 minutes to facilitate liquefaction before semen analysis commenced. Semen parameters, including volume (reference value >1.5 mL), concentration (reference value >15 million sperm/mL), total sperm count (reference value >39 million), percentage of motile sperm (reference value >40%), and percentage of sperm with normal morphology (reference value >4%), were assessed according to the WHO Laboratory Manual for the Examination and Processing of Human Semen (5th Edition, 2010) [39]. Sperm morphology was evaluated using a Diff-Quick staining kit (RS Medical, Ravan Sazeh Co., Iran) following the manufacturer’s guidelines. To determine sperm concentration and motility, a 10 µL semen sample was placed in a Neubauer chamber. Sperm motility was assessed according to the WHO criteria using a phase-contrast microscope at 200x magnification. A semen sample was classified as abnormal if at least one of the semen parameters (volume, concentration, motility, or morphology) fell outside the WHO reference limits mentioned above. All analyses were performed by experienced technicians, and external quality control was maintained throughout the study by cross-checking results with other reference laboratories.

### Biochemical assessments

Blood and semen samples were collected on the same day for all participants. Blood samples (approximately 10 mL) were collected in the morning by a trained phlebotomist, following an overnight fast. Blood was drawn from an antecubital vein using a sterile venipuncture technique and collected in vacutainer tubes containing EDTA anticoagulant. Samples were centrifuged at 4°C for 15 minutes at 2500 x g to separate plasma from red blood cells (RBCs). The separated plasma aliquots were stored at −80°C until further analysis. After liquefaction, the samples were centrifuged at 3000 × g for 15 minutes at 4°C to separate the seminal plasma from the sperm pellet. The resulting cell-free seminal plasma, containing the target biomarkers, was carefully pipetted into separate cryovials and stored at −80°C until further analysis.

### TNF-α levels in seminal plasma and blood serum

TNF-α levels in both serum and seminal plasma were determined using a colorimetric competitive enzyme-linked immunosorbent assay (ELISA) method with a human TNF-α ELISA kit (KIT10602, Sino Biological Inc., Beijing, China), with a sensitivity of 2.84 pg/mL, following the manufacturer’s protocol.

### AGE levels in seminal plasma and blood serum

The levels of AGEs in seminal plasma and blood serum were measured using a human AGE ELISA Kit (E0003Hu, Eastbiopharm, Shanghai Crystal day Biotech Co., Ltd.) with a sensitivity of 9.8 ng/mol in accordance with the manufacturer’s protocol.

### 8-OHdG levels in seminal fluid plasma

Seminal plasma levels of 8-OHdG were measured using a colorimetric competitive ELISA (E202001190601, Eastbiopharm, Shanghai Crystal Day Biotech Co., Ltd.) with a sensitivity of 0.94 ng/mL, in accordance with the manufacturer’s instructions.

### TAC levels in seminal plasma and blood serum

The level of TAC in seminal fluid and blood serum was measured using a highly sensitive kit provided by the KiaZist company (KiaZist, Iran), with a sensitivity of 20 nmol/mL according to the manufacturer’s instructions.

### MDA levels in seminal fluid plasma

Malondialdehyde (MDA), the final product of lipid peroxidation, reacts with thiobarbituric acid (TBA) to create a red-colored complex that absorbs light at 535 nanometers. MDA levels were quantified using a commercially available lipid peroxidation (LPO) assay kit (Nalondi-Lipid Peroxidation Assay Kit-MDA, NS-15022, NS-15023; Navand Salamat Co., Iran) with a sensitivity of 10 μM, according to the manufacturer’s protocol.

### Statistical analysis

Independent-samples t-tests were employed to compare demographic characteristics, including age, BMI, and WHR, as well as semen parameters and biochemical parameters, between men with normal and abnormal semen. Participants were categorized into groups based on quartiles of their dTAC, AHEI, and DII scores. Quartile cutoffs were determined based on the natural distribution of the data in the study population. The normality of the continuous variables was assessed using the Shapiro–Wilk test. Normal variables were presented as mean ± standard deviation (SD) and compared using Student’s t-test or one-way ANOVA. Levene’s test for equality of variances was used to assess the homogeneity of variances. The independent samples t-test was used to compare groups with normal and abnormal semen parameters for demographic characteristics, energy intake, and initial SA results. One-way ANOVA with Tukey’s post hoc test for multiple comparisons was used to analyze the relationship between dietary scores and markers like semen TAC and TNF-α levels. Effect sizes were calculated using the General Linear Model (GLM) to allow precise quantification of the observed effects. Statistical analyses were performed using SPSS software, version 22.0 (IBM Corp., Chicago, IL, USA). A P-value less than 0.05 was considered statistically significant.

### Ethical approval

The study protocol received ethical approval from the Deputy of the Research and Ethics Committee of Tehran University of Medical Sciences (TUMS) on September 26, 2020 (approval code: IR.TUMS.MEDICINE.REC.1399.548). The study was conducted in accordance with the Declaration of Helsinki, the International Council for Harmonisation (ICH) guidelines for Good Clinical Practice, and all applicable regulatory requirements. Confidentiality and privacy of all patient data were strictly maintained throughout the study.

## Results

### Baseline characteristics and semen parameters

Table 1 summarizes the demographic characteristics (age, BMI, WHR, and total energy intake [the amount of energy from food and drinks consumed in a 24-hour period, measured in kilocalories (kcal)]) and semen parameters of the study population (n = 90 infertile men) stratified by normal (n = 44) and abnormal (n = 46) SA. No significant differences were observed in age, BMI, WHR, or total energy intake between groups.

**Table 1:** Demographic characteristics, energy intake, and semen parameters of the study participants.

	Total population of infertile men (n = 90)	Total population		P-value
		Normal Semen (n = 44)	Abnormal Semen (n = 46)	
**Characteristics**				
**Age** (years)	35.28 ± 4.93	34.76 ± 5.27	35.54 ± 4.76	0.40
**BMI** (kg/m^2^)	27.19 ± 3.66	27.52 ± 3.31	27.03 ± 3.85	0.50
**WHR**	0.89 ± 0.02	0.89 ± 0.02	0.89 ± 0.02	0.90
**Total energy** **intake (**kcal/d)	2997.79 ± 985.96	3006.46 ± 996.46	2993.45 ± 986.52	0.90
**Semen parameters**				
**Concentration** (×10^6^/ml)	21.13 ± 15.83	24.23 ± 9.59	19.61 ± 18.02	0.06
**Total motility (%)**	39.47 ± 21.78	53.18 ± 17.92	32.61 ± 20.33	<0.001***
**Normal morphology (%)**	2.86 ± 1.62	4.51 ± 0.79	2.04 ± 1.26	<0.001***
**Volume (ml)**	2.67 ± 1.01	2.66 ± 0.77	2.68 ± 1.12	0.89

Values were tested with independent Student’s t-test and presented as mean ± SD. BMI, Body Mass Index; WHR, waist-to-hip ratio. ***P < 0.001.

Sperm concentration in the normal semen group (24.2 ± 9.59 × 10^6^/ml) and abnormal semen group (19.6 ± 18.02 × 10^6^/ml) did not differ significantly (p = 0.06). However, statistically significant differences were found in total motility (p < 0.001) and normal morphology (p < 0.001) between groups. Men with normal semen exhibited significantly higher values for both parameters (total motility: 53.18 ± 17.92%; normal morphology: 4.51 ± 0.79%) than those with abnormal semen parameters (total motility: 32.61 ± 20.33%; normal morphology: 2.04 ± 1.26%). Semen volume did not differ significantly between groups (p = 0.89).

### Biochemical parameters

**Fig 1:**
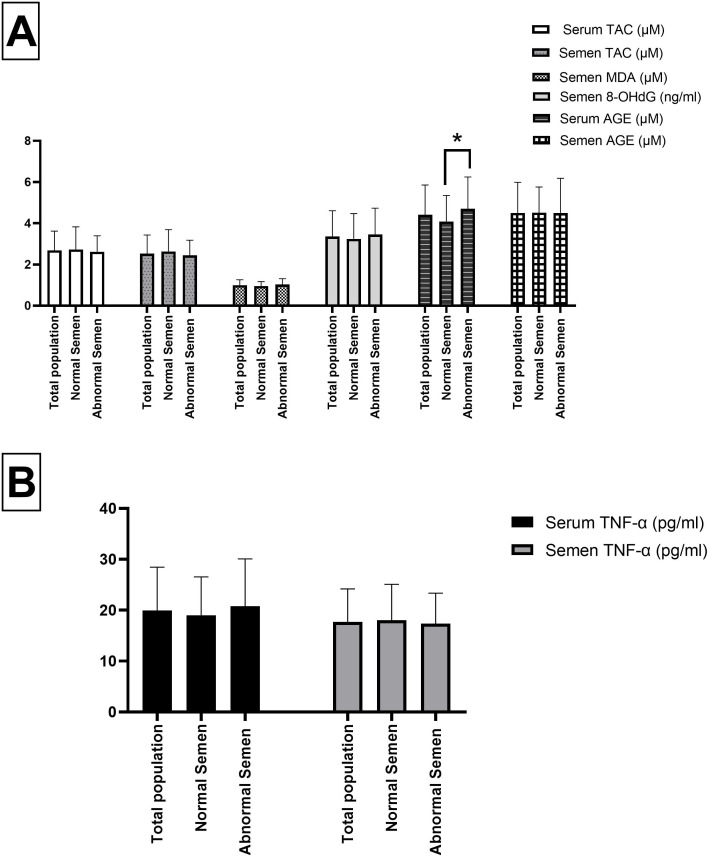
Comparison of biochemical parameters between infertile men with normal and abnormal semen. **A** Comparison of biochemical markers of oxidative stress and)**B**(inflammation in infertile men with normal semen parameters (n = 44) and those with abnormal semen parameters (n = 46). TAC: Total antioxidant capacity; MDA: Malondialdehyde; 8-OHdG: 8-hydroxy-2’-deoxyguanosine; AGE: Advanced glycation end products; TNF-α: Tumor necrosis factor alpha. Data are presented as mean ± SD. Differences between groups were analyzed using the independent Student’s t-test. A p-value < 0.05 was considered statistically significant (* indicates significance).

Fig 1 shows the biochemical parameters measured in serum and semen. Serum AGE was significantly higher in the abnormal semen group (4.7 ± 1.55) compared to the normal semen group (4.09 ± 1.26) (p = 0.04). No significant differences were found in serum or seminal levels of TNF-α, TAC, or MDA. While semen MDA levels were slightly higher in the abnormal semen group (1.03 ± 0.28) compared to the normal semen group (0.95 ± 0.22), the difference was not statistically significant (p = 0.09). Similarly, seminal 8-OHdG levels did not differ significantly between groups (p = 0.41).

### Dietary scores and OS markers

Table 2 shows the serum and seminal TAC and TNF-α levels within quartiles of the dietary scores (dTAC, AHEI, and DII). Quartile analysis revealed that higher quartiles of AHEI and dTAC indicated healthier dietary patterns and greater dietary antioxidant levels, while lower quartiles of DII corresponded to reduced dietary inflammation. No statistically significant differences were found in TAC or TNF-α levels across any dietary score categories. One-way ANOVA revealed no significant differences in serum or seminal OS markers (MDA, 8-OHdG, and AGE) across quartiles of dTAC and DII scores. The AHEI quartiles revealed a significant difference in seminal AGE values between the first quartile and the third and fourth quartiles (p = 0.02 and p = 0.004, respectively; Bonferroni-adjusted p = 0.0033 and p = 0.0006), with a large effect size (partial eta-squared = 0.489). Notably, the trend across AGE quartiles and semen parameters appeared to follow a J-shaped pattern, with the second and third quartiles showing lower values compared to both the first and fourth.However, no significant differences were observed in serum MDA, 8-OHdG, or AGE.

**Table 2:** Serum and Seminal Total Antioxidant Capacity (TAC) and Tumor Necrosis Factor Alpha (TNF-α) Levels across Quartiles of dTAC, AHEI, and DII Scores.

	Serum TAC	Semen TAC	Serum TNF-α	Semen TNF-α	Semen MDA	Semen 8-OHdG	Serum AGE	Semen AGE
**dTAC**								
**Q1** (5.29-18.69)	2.72 ± 0.83	2.77 ± 1.12	17.58 ± 8.14	17.03 ± 5.39	0.99 ± 0.25	3.34 ± 1.35	4.56 ± 1.58	4.93 ± 1.78
**Q2** (19.31-42.89)	2.63 ± 0.90	2.67 ± 0.98	20.41 ± 8.27	17.96 ± 5.69	1.02 ± 0.28	3.36 ± 1.05	4.26 ± 1.45	4.16 ± 1.20
**Q3** (42.93-155.89)	2.55 ± 1.01	2.28 ± 0.61	19.80 ± 7.76	18.47 ± 7.83	1.03 ± 0.29	3.50 ± 1.17	4.23 ± 1.40	4.44 ± 1.43
**Q4** (164.31-1012.36)	2.83 ± 1.05	2.38 ± 0.77	22.77 ± 9.83	17.21 ± 7.11	0.97 ± 0.21	3.20 ± 1.45	4.6 ± 1.40	4.34 ± 1.40
**P-value**	0.70	0.20	0.20	0.80	0.80	0.80	0.70	0.30
**AHEI**								
**Q1** (29-42)	2.62 ± 0.96	2.59 ± 0.95	20.27 ± 8.56	16.57 ± 5.97	0.98 ± 0.27	3.13 ± 1.22	4.50 ± 1.57	5.02 ± 1.67 ^a,b^
**Q2** (43-49)	2.60 ± 0.83	2.69 ± 0.96	18.28 ± 7.97	17.20 ± 6.41	1.03 ± 0.22	3.41 ± 1.35	4.45 ± 1.29	4.08 ± 1.02
**Q3** (50-56)	2.6 ± 0.78	2.67 ± 0.98	19.03 ± 9.23	18.48 ± 6.88	1.03 ± 0.30	3.51 ± 1.17	4.269 ± 1.37	3.74 ± 1.32 ^a^
**Q4** (57-73)	2.85 ± 1.14	2.21 ± 0.70	21.87 ± 8.34	18.13 ± 6.84	0.98 ± 0.24	3.35 ± 1.31	4.46 ± 1.61	5.14 ± 1.46 ^b^
**P-value**	0.70	0.20	0.50	0.70	0.80	0.80	0.95	0.002**
**DII**								
**Q1** (−5.16- −3.75)	2.87 ± 1.24	2.28 ± 0.78	18.40 ± 7.60	15.18 ± 7.32	0.98 ± 0.27	2.95 ± 1.25	3.90 ± 1.20	4.88 ± 1.31
**Q2** (−3.72- −3.06)	2.56 ± 0.85	2.48 ± 0.76	19.97 ± 9.65	18.93 ± 6.52	1.00 ± 0.30	3.53 ± 1.21	4.61 ± 1.67	4.16 ± 1.83
**Q3** (-3.05- −2.38)	2.69 ± 0.96	2.56 ± 1.10	21.13 ± 7.33	16.74 ± 6.94	1.03 ± 0.23	3.64 ± 1.22	4.62 ± 1.40	4.66 ± 1.25
**Q4** (−2.37- −0.71)	2.66 ± 0.79	2.72 ± 0.92	19.81 ± 9.26	16.17 ± 5.23	1.00 ± 0.22	3.17 ± 1.29	4.34 ± 1.40	4.44 ± 1.43
**P-value**	0.78	0.50	0.81	0.32	0.91	0.28	0.40	0.40

Differences across quartiles were tested using one-way ANOVA followed by Tukey’s post hoc test. TAC; total antioxidant capacity, MDA; malondialdehyde, 8-OHdG; 8-hydroxy-2’-deoxyguanosine, AGE; advanced glycation end products, TNF-α; tumor necrosis factor alpha. Data are presented as mean ± SD. A p-value < 0.05 is considered significant. ^a^ Statistically significant difference. ^**^P < 0.01. a: P = 0.02 and b: P = 0.004.

## Discussion

In this cross-sectional study conducted at two major referral centers, involving 90 infertile men who presented to infertility clinics from 2020 to 2022, we observed that serum AGE levels were significantly higher in the abnormal semen group compared to the normal semen group. However, no significant differences appeared in other serum or semen biochemical parameters. There were also no statistically significant differences across categories of the dTAC, AHEI, and DII scores in TAC and TNF-α levels. However, a significant difference was observed in seminal AGE values within quartiles of the AHEI score.

OS is a well-established contributor to idiopathic male infertility, with studies indicating that spermatozoa exhibiting morphological abnormalities are more likely to generate excessive reactive oxygen species (ROS) and have diminished antioxidant capacity [52]. OS can induce DNA damage in both mitochondria and the nucleus, and it also affects the sperm epigenome. Consequently, this DNA damage can lead to infertility, recurrent spontaneous abortion, adverse pregnancy outcomes, and an elevated risk of diseases in the offspring [53]. Additionally, men with idiopathic infertility often show an imbalance between ROS levels and antioxidant capacity compared to fertile males [54]. Effective strategies to mitigate OS remain elusive [55]. We found that there were no significant differences in OS markers (TAC, MDA, 8-OHdG) in serum and seminal plasma between infertile men with normal and abnormal semen. This finding is expected, as all participants were infertile, and oxidative stress may be a common underlying factor regardless of semen quality. While OS is a key contributor to idiopathic infertility, other factors such as hormonal or genetic abnormalities may also play roles. Future studies with larger samples and broader OS marker panels (e.g., CAT, GPx, SOD) are warranted to clarify the complex link between OS and male infertility.

Several studies have reported associations between reduced TAC in seminal plasma and male infertility [56–59]. Several studies have reported a positive relationship between seminal total antioxidant capacity (TAC) and sperm quality parameters. Eroglu et al. [60] demonstrated a significant correlation between seminal TAC and sperm concentration, motility, and morphology in men with idiopathic infertility. Consistently, Subramanian et al. [61] reported a positive correlation between seminal TAC levels and sperm concentration, motility, and morphology, while Giulini et al. [58] also reported reduced seminal TAC levels in varicocele patients with impaired semen quality, reinforcing the potential link between oxidative balance and sperm motility. In contrast, our study observed no significant differences in serum or seminal TAC levels between infertile men with normal and abnormal semen parameters. This may indicate that oxidative stress is a shared underlying factor among infertile men regardless of semen quality. Further studies assessing a broader range of oxidative stress biomarkers (e.g., TOS, OSI, TAS, PAB, GSH-Px, PON, AOPP) are required to provide a more comprehensive understanding of redox balance in male infertility.

The Western diet—characterized by elevated DII scores—is considered pro-inflammatory and is implicated in the upregulation of several genes (e.g., IL-6, IL-1, TNF-α) involved in pro-inflammatory pathways and is therefore regarded as a predisposing factor for male infertility. In contrast, the Mediterranean diet is associated with enhanced sperm quality and improved fertility potential [42]. Inflammation can lead to male reproductive disorders, irregular menstrual cycles, difficulties with implantation, and other reproductive issues [62]. The detrimental effects of inflammation on spermatogenesis are primarily attributed to OS induced by elevated ROS and pro-inflammatory cytokines, leading to germ cell damage and impaired steroidogenesis [4]. Furthermore, the hypothalamic-pituitary-gonadal (HPG) axis and spermatogenesis are adversely impacted by inflammatory cytokines [17]. Consequently, dietary strategies aimed at mitigating inflammation hold promise for improving fertility outcomes [62]. Nonetheless, in our study, we found no differences between groups in serum and seminal levels of TNF-α. Consistent with our findings, Eggert-Kruse et al. [63] also reported no significant association between seminal TNF-α and IL-1β levels with semen quality, sperm fertilizing capacity, or subsequent fertility in subfertile men. Nevertheless, Shukla et al. [64] reported a significantly higher prevalence of TNF-α gene mutation among infertile men with asthenozoospermia compared to fertile controls, suggesting a potential role for this cytokine in the pathophysiology of male infertility. Variations in TNF-α levels and related genetic factors may influence inflammatory responses in the male reproductive system. Further studies in larger and diverse populations are needed to clarify the specific contribution of TNF-α to semen quality.

There were no statistically significant differences between categories of the dTAC, AHEI, and DII scores in TAC and TNF-α levels. Similarly, Salas-Huetos et al. [65] reported no significant associations between men’s adherence to several healthy dietary patterns—including the AHEI and Mediterranean diet scores—and either semen quality or ART outcomes in a large prospective cohort of infertile couples. In contrast, the recent Led-Fertyl study by Davila-Cordova et al. (2025) [66], led by Salas-Huetos, demonstrated that greater adherence to healthy dietary patterns—particularly the Mediterranean and healthful plant-based diets—was associated with higher sperm count and motility, whereas adherence to Western and unhealthful diets was inversely related to semen quality. Moradi et al. [67] reported higher MDA and DII levels in patients with non-alcoholic fatty liver disease compared to healthy controls, while TAC was higher in the latter. In healthy individuals, TAC showed a negative and MDA a positive correlation with DII, suggesting that underlying health status may influence the relationship between dietary inflammation and oxidative stress markers. Taken together, these results reinforce the notion that overall diet quality may influence male reproductive potential, although the strength of such associations can vary depending on clinical status and underlying oxidative or inflammatory conditions. Overall, these findings suggest that while healthy diets are beneficial, their measurable effects on reproductive biomarkers may be modest or masked by other clinical and lifestyle factors.

Building on our previous findings [38], this study examined seminal oxidative and inflammatory markers and found that dietary patterns may influence semen quality, particularly through modulation of AGE levels. Although no direct associations with conventional semen parameters were observed, the results suggest a potential role for diet in male reproductive health. There were no notable differences across categories of the dTAC and DII scores in serum and seminal plasma levels of OS markers. The AHEI quartiles showed a similar pattern in serum MDA, 8OHdG, and AGE. However, there was a significant difference in seminal AGE values across quartiles of the AHEI score. This finding suggests a potential link between a healthy dietary pattern (as measured by AHEI) and seminal AGE levels, highlighting the importance of diet in male reproductive health. AGEs are critical contributors to oxidative stress and inflammation, which can significantly affect male fertility. By binding to their receptor RAGE (Receptor for Advanced Glycation End-products), AGEs trigger signaling cascades that increase ROS production and inflammatory responses. Studies in diabetic men and rodent models have demonstrated that elevated AGEs correlate with increased MDA, reduced TAC, and impaired sperm and testicular function [68–70]. Moreover, elevated levels of RAGE and MDA were observed in the testes of the mice. In our study, serum AGE levels were significantly elevated in the abnormal semen group compared to the normal semen group. Nevertheless, there were no significant differences in seminal AGE levels between groups. Differences in the concentrations of biochemical markers between seminal plasma and serum may result from the specialized secretions of the testes, epididymis, and accessory glands. The unique environment required to support sperm function and metabolism may also contribute to these differences. Sperm function may be impaired by oxidative damage to the sperm membrane, DNA fragmentation, and mitochondrial dysfunction caused by the binding of AGEs to their receptors. Furthermore, AGE-mediated inflammation can disrupt the testicular microenvironment, negatively impacting spermatogenesis and overall semen quality. Given these differences, it is plausible that dietary patterns influencing AGE accumulation may indirectly affect semen parameters through these biological pathways. Furthermore, understanding AGE-RAGE signaling in the testicular microenvironment may offer insights into improving male fertility [68,71].

A healthy diet may ameliorate OS [72]. Mirmiran et al. [73] found a significant positive link between a healthy diet and serum TAC levels, and a negative association between a healthy diet and serum MDA. We found no associations between dietary pattern scores and serum or seminal OS markers. We also found no significant differences in serum MDA levels across the different categories of the dTAC score. However, in a study on postmenopausal women, Abshirini et al. [74] reported a reciprocal relationship between dTAC and serum MDA level in postmenopausal women. Furthermore, serum TAC levels were significantly higher in participants within the second tertile of dTAC. However, no significant relationship was detected between dTAC and serum SOD concentration. Overall, these findings support the potential of antioxidant-rich diets to improve seminal oxidative balance

This study used hospital-based data collected over two years to comprehensively assess the association between multiple dietary quality scores and semen biochemical parameters, allowing a comprehensive evaluation of diet and semen quality in infertile men with normal and abnormal semen undergoing assisted reproduction at TUMS-affiliated clinics. The study population was carefully selected for similarity in age, ethnicity, and clinical variables, enhancing the generalizability of our findings to Iranian infertile men. However, it is crucial to note that there are limitations to our study. We included only men with idiopathic infertility and normal physical and medical examinations, and dietary intake assessment relied on self-reported recall, which may introduce measurement error. The cross-sectional design limits causal inferences, and the modest sample size may restrict generalizability. The absence of a healthy fertile control group could also limit comparisons and may affect the external generalizability of the findings; however, stratification within idiopathic infertile men allowed us to explore lifestyle-related variations in semen quality in a clinically homogeneous population. Despite these limitations, this study provides valuable insights and lays the groundwork for larger, longitudinal, multi-center investigations to further clarify the impact of diet on male reproductive health.

## Conclusions

Our study aimed to clarify the impact of diet on male fertility, particularly highlighting how the intake of AGEs is associated with abnormal seminal parameters. The results suggest that a healthy dietary pattern, as measured by the AHEI, could positively influence seminal AGE levels. Thus, promoting a healthy dietary pattern could be an effective strategy to improve reproductive health in infertile men. The study concluded that adopting diets that reduce inflammation and increase antioxidant intake might help enhance sperm quality.

### Future directions and practical implications

While larger studies are needed to confirm these findings, promoting healthy dietary patterns low in AGEs and rich in antioxidants may be a useful strategy to improve male fertility. Future research should employ a multidimensional approach, considering hormonal, genetic, and environmental factors alongside dietary interventions. Randomized controlled trials can further evaluate the effectiveness of dietary modifications in optimizing male reproductive health.

## List of abbreviations:

WHO: World Health Organization
BMI: Body Mass Index
WHR: Waist-to-Hip Ratio
FFQ: Food Frequency Questionnaire
ELISA: Enzyme-Linked Immunosorbent Assay
AGE: Advanced Glycation End Product
TNF-α: Tumor Necrosis Factor-alpha
TAC: Total Antioxidant Capacity
dTAC: Dietary Total Antioxidant Capacity
8-OHdG: 8-hydroxy-2’-deoxyguanosine
DII: Dietary Inflammatory Index
MDA: Malondialdehyde
OS: Oxidative Stress
OSI: Oxidative Stress Index
ROS: Reactive Oxygen Species
SA: Semen Analysis
AHEI: Alternative Healthy Eating Index
TLGS: Tehran Lipid and Glucose Study
PUFAs: Polyunsaturated Fatty Acids
